# Evidence on the effectiveness and equity of population-based policies to reduce the burden of type 2 diabetes: a narrative review

**DOI:** 10.1007/s00125-024-06330-1

**Published:** 2024-12-02

**Authors:** Joreintje D. Mackenbach, Josine M. Stuber, Joline W. J. Beulens

**Affiliations:** 1 Amsterdam UMC location Vrije Universiteit, Epidemiology and Data Science, Amsterdam, the Netherlands; 2 Amsterdam Public Health Research Institute, Amsterdam, the Netherlands; 3 Upstream Team, Amsterdam, the Netherlands

**Keywords:** Agency, Obesity, Population-level approaches, Prevention, Review, WHO Best Buys

## Abstract

There is increasing evidence for the effectiveness of population-based policies to reduce the burden of type 2 diabetes. Yet, there are concerns about the equity effects of some policies, whereby socioeconomically disadvantaged populations are not reached or are adversely affected. There is a lack of knowledge on the effectiveness and equity of policies that are both population based (i.e. targeting both at-risk and low-risk populations) and low agency (i.e. not requiring personal resources to benefit from the policy). In this narrative review, we selected 16 policies that were both population based and low agency and reviewed the evidence on their effectiveness and equity. Substantial evidence suggests that fruit and vegetable subsidies, unhealthy food taxes, mass media campaigns, and school nutrition and physical activity education are effective in promoting healthier lifestyle behaviours. Less evidence was available for mandatory food reformulation, reduced portion sizes, marketing restrictions and restriction of availability and promotion of unhealthy products, although the available evidence suggested that these policies were effective in reducing unhealthy food choices. Effects could rarely be quantified across different studies due to substantial heterogeneity. There is an overall lack of evidence on equity effects of population-based policies, although available studies mostly concluded that the policies had favourable equity effects, with the exception of food-labelling policies. Each of the policies is likely to have a relatively modest effect on population-level diabetes risks, which emphasises the importance of combining different policy measures. Future research should consider the type of evidence needed to demonstrate the real-world effectiveness and equity of population-based diabetes prevention policies.

**Graphical Abstract:**

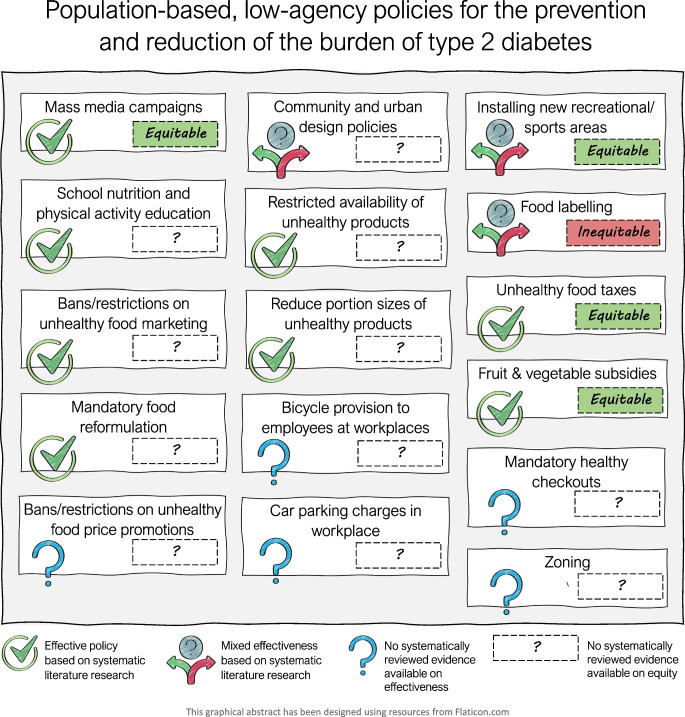

**Supplementary Information:**

The online version contains a slideset of the figures for download available at 10.1007/s00125-024-06330-1.

## Introduction

Type 2 diabetes is largely preventable through lifestyle modifications. RCTs of intensive lifestyle interventions delivered to individuals at high risk of type 2 diabetes suggest 45% risk reductions at intervention cessation [1]. Most intensive lifestyle interventions can be considered very cost effective [2, 3]. However, because they generally reach a highly educated and motivated selection of individuals at risk [4–7], their impact on a population level has been estimated as low [8, 9].

**Fig. 1:**
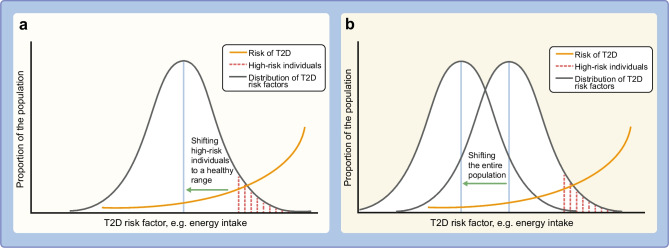
Illustration of Geoffrey Rose’s prevention paradox. Geoffrey Rose’s prevention paradox [10] states that only a minority of disease cases originates from a high-risk population, with the majority originating from low-/moderate-risk populations. Therefore, policies that target whole populations (rather than just high-risk individuals) are expected to be more effective. (**a**) Graph demonstrating that reducing risk in high-risk individuals has only a small influence on the distribution of risk factors and, thus, the health of most people. (**b**) Graph demonstrating that a population-based approach that reduces risk factors in the whole population shifts the entire distribution of the risk in the population in a favourable way. Blue vertical lines, mean value of the risk factor of interest. T2D, type 2 diabetes. This figure is available as part of a downloadable slideset

Geoffrey Rose’s prevention paradox [10] states that the majority of disease cases originates from a population at low or moderate disease risk, and only a minority of cases originates from a high-risk population (Fig. 1). This implies that most gains can be expected from policies targeting the population at low or moderate risk, i.e. the population as a whole instead of only the high-risk individuals. Increasing evidence is available on the efficacy of population-based policies, such as mass media campaigns, healthy food subsidies, and park and playground renovations [11].

There are, however, concerns about the equity effects of some population-based policies. Like with approaches targeting high-risk populations, socioeconomically disadvantaged populations may not be reached or may be adversely affected by population-based policies [12]. If so, population-based policies may inadvertently widen health inequalities. This may particularly be the case for policies requiring individual agency (i.e. personal resources) in order to benefit. For instance, media campaigns require individuals to take multiple steps in order to benefit from them: being aware of the campaign, understanding the campaign, remembering and prioritising it, initiating behaviour change and maintaining the behaviour change in the long term. The behaviour change must additionally be substantial enough to translate into decreased type 2 diabetes risk. In each step along the way to disease prevention, individuals may revert to old habits, resulting in only a selection of the population that ultimately experiences health benefits. Lifestyle interventions and population-based policies that require use of personal resources have been coined ‘highly agentic’ [13]. Low-agency policies are those that require few personal resources to experience beneficial effects [13], such as a mandated sugar reduction in beverages to which all individuals purchasing beverages are subjected.

It has been hypothesised that low-agency policies reach a wider population and are more equitable. In this narrative review, we summarised the availability and quality of the evidence for the efficacy, cost effectiveness and equity of policies aimed at preventing or reducing the burden of type 2 diabetes that are both population based and low agency.

## Approach

We constructed a list of generic policies based on an exploratory literature search and the list of ‘best buys to tackle non-communicable diseases’ by the WHO [14]. We focused on policies directly targeting type 2 diabetes prevention but also policies targeting the intermediate risk factors, obesity, physical activity and dietary intake. We classified policies according to whether they are high risk or population based, and whether they were high or low agency with the Demands for Population Health Interventions (Depth) framework as guidance [15]. Because we evaluated general types of policies rather than specific interventions, it was not possible to follow the complete Depth procedure. Instead, we identified the agentic demand of the policy based on: (1) the exposure (way of getting in contact with the intervention), categorised as active or passive; (2) the mechanism of action (way of components altering the outcome), categorised as socio-cultural, cognitive, financial, physical–environmental and biomedical; and (3) the level of engagement (degree to which recipients are required to be aware of or interact with the component in order to benefit as intended), categorised as active or passive. For the purpose of this narrative review, we regarded policies with passive exposure as low-agency policies, although this means that some of the considered policies still need a certain level of active engagement of recipients to benefit from them. We conducted a literature search in PubMed on 30 April 2024 for systematic reviews and meta-analyses published between 2014 and 2024 on the efficacy, cost effectiveness and (socioeconomic) equity of population-level and low-agency policies. We also summarise the quality of the evidence as reported by the authors of the reviews. If no systematic reviews were available, we summarised recent evidence from primary studies.

## Findings

In Table 1, we classified the identified type 2 diabetes prevention policies according to their mechanism of action and whether they can be regarded as high risk or population based. Lifestyle and screening programmes that are only offered to and reimbursed for individuals with a risk indication for type 2 diabetes (e.g. those with obesity) were classified as high-risk policies. In Table 2, we list all the population-based policies according to whether the exposure to and engagement with the policy is active or passive. Active exposure refers to a situation where recipients have to change their usual daily activities to come into contact with the policy; for instance, when employers offer exercise equipment to their employees, but the employees have to organise their workday and breaks differently to benefit from it. Active engagement refers to a situation where recipients need to be aware of the mechanism of action and interact with it in order to benefit. An example of this is food labelling, whereby individuals need to notice and interpret the labels and understand that the provided information could guide their choice. We regarded the 16 policies classified as having passive exposure as low-agency policies.

**Table 1:** Types of interventions and policies available for the prevention and reduction in the burden of type 2 diabetes according to their mechanism of action

Mechanism of action/type of intervention or policy	High-risk or population-based policy?
Socio-cultural policies (aim to change a community or society’s attitudes, beliefs, norms and values related to the intended behaviour)	
Mass media campaigns to promote healthy nutrition and PA behaviours, e.g. TV campaigns, risk communication campaigns, healthy lifestyle promotion campaigns	Population-based
School nutrition and PA education	Population-based
Physical–environmental policies (aim to change the availability, accessibility, safety, placement or properties of infrastructure, facilities, objects or stimuli in the wider environment)	
Regional or national workplace stair-use policy, or provision of PA equipment or activity during break times	Population-based
Community-based lifestyle approaches	Population-based
Community and urban design policies (e.g. community regeneration to improve walkability, bike paths)	Population-based
Installing new recreational/sports areas	Population-based
Food environment policies	
Bans or restrictions on unhealthy ingredients or products (mandatory food reformulation)	Population-based
Bans or restrictions on unhealthy product marketing	Population-based
Restricted availability of unhealthy products	Population-based
Mandatory healthy checkouts	Population-based
Reduced portion size of unhealthy products	Population-based
Zoning (restricting unhealthy food outlets, e.g. around schools)	Population-based
Financial policies (aim to change the relative monetary cost of intended behaviours)	
Food assistance programmes	High-risk^a^
Incentivising PA via healthcare insurance	Population-based
Provision of food vouchers for healthy products	Population-based
Unhealthy food taxes (e.g. taxes on SSBs, alcohol)	Population-based
Fruit and vegetable subsidies	Population-based
Bans or restrictions on unhealthy food price promotions	Population-based
National level policies on bicycle provision to employees at workplaces	Population-based
Car parking charges in workplaces	Population-based
Cognitive policies (aim to change an individual’s knowledge, attitudes, beliefs or skills concerning the intended behaviour)	
Lifestyle programmes (e.g. combined lifestyle programmes, digital lifestyle counselling, intensive lifestyle programmes)	High-risk
Population-level provision of wearables for PA monitoring (e.g. the National Steps Challenge programme in Singapore [16])	Population-based
Mobile or email messages aiming to promote individual beliefs on healthy diets	Population-based
Food labelling (e.g. mandatory, voluntary, front-of-pack, menu labelling)	Population-based
Biomedical policies (involve drug or medical techniques that aim to alter the intended behaviour or biological systems)	
National screening programmes and screening by the GP (e.g. for impaired glucose tolerance, GDM or diabetes complications)	High-risk
Reimbursement for medication after screening for prediabetes (e.g. weight-lowering medications, insulin sensitisers, α-glucosidase inhibitors)	High-risk
Reimbursement for bariatric surgery	High-risk

^a^High risk in this case is defined as high risk of socioeconomic security

GDM, gestational diabetes; GP, general practitioner; PA, physical activity; TV, television

**Table 2:** Types of population-based policies available for the prevention and reduction in the burden of type 2 diabetes according to their agentic demand based on the mode of exposure, mechanisms of action and the type of engagement required

Exposure	Mechanism of action				Engagement
	Socio-cultural	Physical–environmental	Financial	Cognitive	
Active^a^		• Regional or national workplace stair-use policy, or provision of PA equipment or activity during break times • Community-based lifestyle approaches	• Incentivising PA via healthcare insurance	• Population-level provision of wearables for PA monitoring	Active^c^
Active^a^			• Provision of food vouchers for healthy products	• Mobile or email messages aiming to promote individual beliefs on healthy diets	Passive^d^
Passive^b^	• Mass media campaigns to promote healthy nutrition and PA behaviours • School nutrition and PA education	• Community and urban design policies • Installing new recreational/sports areas • Zoning • Mandatory healthy checkouts	• Unhealthy food taxes • Fruit and vegetable subsidies • Bans or restrictions on unhealthy food price promotions • National level policies on bicycle provision to employees at workplaces • Car parking charges in workplaces	• Food labelling	Active^c^
Passive^b^		• Bans or restrictions on unhealthy ingredients or products (mandatory food reformulation) • Bans or restrictions on unhealthy product marketing • Restricted availability of unhealthy products • Reduced portion size of unhealthy products			Passive^d^

Note that none of the biomedical policies identified were deemed to be population-based; thus, policies with a biomedical mechanism of action have not been included in this table

^a^Active exposure requires recipients to change their existing daily activities or initiate new activities to come into contact with the intervention component

^b^Passive exposure does not require recipients to make a change from existing daily activities to come into contact with the intervention component

^c^Active engagement requires recipients to be aware of the mechanism of action of the policy/intervention and have purposive interaction with it in order to benefit

^d^Passive engagement does not require recipients to be aware of or interact with the mechanism of action of the policy/intervention in order to benefit. It is possible for recipients to be aware and interact with the mechanism of action, but not a necessity

PA, physical activity

**Fig. 2:**
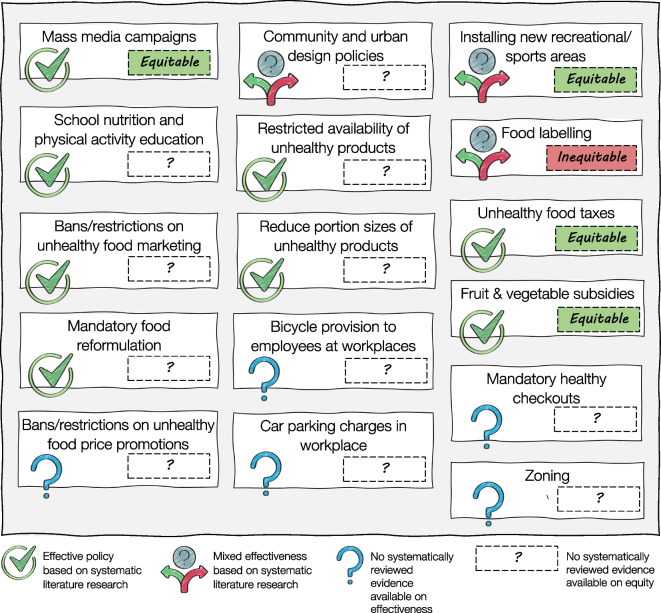
Population-based, low-agency policies for the prevention and reduction of the burden of type 2 diabetes. Illustration of the available evidence for the effectiveness and equity of population-based, low-agency policies for the prevention and reduction of the burden of type 2 diabetes. Figure designed using resources from Flaticon.com. This figure is available as part of a downloadable slideset

Below we discuss the evidence identified for each of the 16 population-based low-agency policies in more detail. The available evidence for the effectiveness, cost-effectiveness and equity effects of the 16 population-based low-agency policies are summarised in Table 3 and Fig. 2.

**Table 3:** Available evidence for the effectiveness, cost effectiveness and equity of population-based, low-agency policies for the prevention and reduction in the burden of type 2 diabetes

Policy	Availability of systematically reviewed evidence	Reported effectiveness	Reported cost effectiveness	Reported equity effects	Reported overall quality of evidence (*n* reviews)
Mass media campaigns to promote healthy nutrition and PA behaviours	Yes [17–21]	Effective in promoting PA and fruit and vegetable intake	Contradictory results	PA mass media campaigns were mostly equitable	High quality (*n*=3), low quality (*n*=1), NR (*n*=1)
School nutrition and PA education	Yes [22–26]	Effective in promoting PA and small positive effects on food consumption and BMI	PA education is mostly cost effective	NR	Mixed (low-quality studies excluded) (*n*=1), low risk of bias (*n*=1), serious bias (*n*=1), NR (*n*=2)
Community and urban design policies	Yes [22, 27–33]	Mixed effects, with likely positive effects of transportation infrastructure on transport-related PA	NR	NR	High quality (*n*=1), moderate quality (*n*=1), low quality (*n*=2), NR (*n*=4)
Installing new recreational/sports areas	Yes [34, 35]	Some effects on PA, fitness, weight loss and T2D-related outcomes	NR	No differential effects observed	Mixed quality (low to high) (*n*=2)
Zoning	No	–	–	–	–
Mandatory healthy checkouts	No	–	–	–	–
Bans or restrictions on unhealthy ingredients or products (mandatory food reformulation)	Yes [36–38]	Effective in reducing energy intake, sugar intake, obesity prevalence, T2D prevalence, and promoting weight loss	NR	NR	Very low quality (*n*=1), NR (*n*=2)
Bans or restrictions on unhealthy product marketing	Yes [39]	Effective in reducing unhealthy purchases in households with children	NR	NR	Low to very low certainty (*n*=1)
Restricted availability of unhealthy products	Yes [40]	Effective in reducing unhealthy food selection and intake	NR	NR	Low certainty (*n*=1)
Reduced portion size of unhealthy products	Yes [41, 42]	Effective in reducing energy intake and promoting weight loss	NR	NR	Moderate quality (*n*=1), NR (*n*=1)
Unhealthy food taxes	Yes [43–53]	Effective in reducing demand and sales of taxed products	NR	Similar or greater effects for socioeconomically disadvantaged vs advantaged groups	Moderate quality (*n*=2), studies met most quality criteria (*n*=1), highly variable study quality (*n*=1), NR (*n*=7)
Fruit and vegetable subsidies	Yes [2, 44, 45, 52–62]	Effective in increasing fruit and vegetable purchases, reducing weight and preventing CVD cases	Contradictory results	Effects seem to be limited to children and women from socioeconomically disadvantaged families	Moderate quality (*n*=4), high risk of bias (*n*=1), quality was variable (*n*=1), quality was poor (*n*=1), NR (*n*=6)^a^
Bans or restrictions on unhealthy food price promotions	No	–	–	–	–
National level policies on bicycle provision to employees at workplaces	No	–	–	–	–
Car parking charges in workplaces	No	–	–	–	–
Food labelling	Yes [63–70]	Mixed effects of front-of-pack labelling on healthier product purchases. Effective in reducing energy intake		Potentially inequitable since socioeconomically disadvantaged populations benefit less from food labelling	NR (*n*=8)

^a^Evidence for policies on fruit and vegetable subsidies originates mostly from studies with high risk of bias and of poor to moderate quality, although reviews of RCTs [58] and prospective studies [56] show similar effects

*NR*, not reported; *PA*, physical activity; *T2D*, type 2 diabetes

### Socio-cultural policies

#### Mass media campaigns to promote healthy nutrition and physical activity behaviours

Two umbrella reviews reported that mass media campaigns are effective in promoting physical activity [17] and fruit and vegetable intake [18]. Mass media campaigns are also likely to be effective among populations with social disadvantages as long as the campaigns are tailored to this population [17]. Moreover, physical activity-targeting mass media campaigns mostly have equitable effects or a better impact on socioeconomically disadvantaged populations [19]. Two reviews reported on the cost effectiveness of mass media campaigns and their findings were contradictory; one systematic review of reviews from 2017 concluded that the quality of the available evidence was high and that the mass media campaigns are highly cost effective [20]. In contrast, in 2022, a systematic review of 25 original studies concluded that the quality of evidence was low to very low, and that the evidence for cost effectiveness was inconclusive [21]. Only one original study was shared among the two reviews [20, 21], thus they largely reviewed different literature.

#### School nutrition and physical activity education

Five systematic or umbrella reviews reported on school nutrition and physical activity education [22–26]. One umbrella review concluded that physical education is effective in promoting physical activity, especially when participation is mandatory, when the physical education is provided by trained teachers and when the lessons are part of a standardised physical education curriculum [22]. A cost-effectiveness review concluded that planned routine physical activities at school are mostly cost effective [23]. Another review reported that school-based cooking programmes increased vegetable intake, although increases were small and the certainty of evidence was very low [24]. Yet, educational nutrition interventions based on behaviour-change theories have positive effects on food consumption in randomised trials [25]. Finally, a systematic review and meta-analysis based on pooled information from 4417 children showed that completing nutrition lessons and physical education as part of the regular school curriculum for a duration of 12 months lowered BMI *z* scores on average by 0.14 (95% CI −0.25, −0.03) [26]. These reviews did not provide evidence on equity effects.

### Physical–environmental policies

#### Community and urban design policies

Eight systematic reviews summarised the effects of urban design interventions or policies [22, 27–33]. New transit infrastructure may decrease total physical activity while increasing transport-related physical activity [27, 28], and new walking and cycling infrastructure is likely to increase physical activity [28, 29]. Urban green-space interventions, such as park renovations, are often effective in promoting physical activity [28, 31, 33] and are most likely to have an effect when combined with a promotion/marketing programme [31]. Improved accessibility to destinations (e.g. improving land use mix) is also associated with increased walking for transportation [28]. Reviews reporting on a variety of urban-form elements (e.g. community-based built environment interventions) found mixed results [22, 28, 30, 32, 33], with around 40% of studies reporting null effects [33]. These reviews did not provide evidence on cost-effectiveness or equity effects.

#### Installing new recreational/sports areas

For outdoor recreational facilities, one review showed some evidence to support that outdoor gyms may improve physical activity, fitness, weight loss and type 2 diabetes-related outcomes [34]. This review reported no consistent evidence for differential usage by age, gender, ethnicity or cultural background. Another review reported that outdoor gyms are mainly used by older adults, but did not report on other sociodemographic differences in usage [35]. These reviews did not provide evidence for cost-effectiveness outcomes.

#### Zoning

No systematic reviews are available for the effects of zoning policies on food purchasing, consumption or health outcomes. Four primary studies on restricting fast-food outlets or promoting healthy food outlets from the USA and UK show either null results or beneficial effects on fast-food outlet density or fruit and vegetable consumption [71–74].

#### Mandatory healthy checkouts

No systematic reviews are available for the effects of healthy checkout policies on food purchasing, consumption or health outcomes. However, the most recent primary studies suggest that healthy checkout policies in grocery stores can increase the purchases of healthier snacks [75–79] and might reduce unhealthier purchases. Moreover, healthy checkout policies may improve nutrition equity since low-income consumers, and Hispanic, non-Hispanic American Indian or Alaska Native, and Black consumers have been found to be more likely to purchase food or beverages from store checkouts [80].

#### Bans or restrictions on unhealthy ingredients or products (mandatory food reformulation)

A systematic review focusing on controlled trials demonstrated that lowering the energy density of foods leads to reductions in daily energy intake and to modest weight loss [36], while another review showed that reducing sugar content of food and beverages significantly reduces sugar intake and body weight [37]. A systematic evaluation of modelling studies [38] showed that a 5% and 25% reduction in sugar content could reduce dietary intake by 16.736 kJ (4 kcal) and 112.968 kJ (27 kcal) per day, respectively (*n*=2 studies [81, 82]), while a sugar reduction of 5–40% or total replacement of sugars with artificial sweeteners could reduce obesity prevalence by 0.2–5% (*n*=3 studies [81, 83, 84]) and type 2 diabetes incidence by 5.8–31.1 cases per 100,000 person-years (*n*=1 study [81]). These reviews did not assess cost effectiveness or equity of the policy. A simulation study suggested that the largest declines in type 2 diabetes incidence following sugar reduction is expected among ethnic minority groups [85].

#### Bans or restrictions on unhealthy product marketing

Evidence regarding the restriction of unhealthy food marketing mostly relies on policies aimed at restricting marketing exposure to children and adolescents. One systematic review suggests that food marketing policies may result in reduced unhealthy food purchases in households with children, with limited evidence on changes in dietary intake [39]. A modelling study has estimated that banning unhealthy food and beverage advertising between 05:30 hours and 21:00 hours could lead to a daily reduction in energy intake by 38.074 kJ (9.1 kcal) (95% CI 2.092, 74.057 kJ [95% CI 0.5, 17.7 kcal]), whereby the highest reductions in energy intake are expected in children who are overweight [86]. This reduction in energy intake is estimated to reduce childhood obesity by 4.6% (95% CI 1.4%, 9.5%) [86]. Cost effectiveness and equity could not be assessed for the policy.

#### Restricted availability of unhealthy products

A systematic review and meta‐analysis of three studies showed that reduced exposure to unhealthy products resulted in a 35.6% reduction in the selection of these products. In addition, reduced exposure led to a moderate reduction in consumption of these products [40]. Efficacy studies suggest that increasing the availability of low-energy products increases the likelihood of their selection [87, 88]. No systematically reviewed evidence on cost effectiveness or equity was available.

#### Reduced portion size of unhealthy products

Two systematic reviews showed that reduced portion sizes reduced daily energy intake by 1046.000–1255.200 kJ (250–300 kcal), both based on *n*=14 studies with intervention durations ranging from 1 day to half a year [41, 42]. Based on meta-regressions, a portion reduction of 418.400 kJ (100 kcal) led to a 58.576 kJ (14 kcal) reduction in daily energy intake, and individuals who were being served reduced portions gained 0.6 kg less weight than those being served larger portions [41]. These reviews did not provide evidence on cost effectiveness or equity.

### Financial policies

#### Unhealthy food taxes

No less than 11 of the systematic reviews identified reported on the effects of food taxes [43–53]. All of these reviews reported that food taxes reduce demand for the taxed products [43–53], with stronger (expected) effects in low- and middle-income countries than in high-income countries [47, 48]. Most reviews concluded that a 15–20% tax rate would be recommended [44, 45, 48, 49], but that substitution effects should be considered to estimate the effects on dietary quality and health outcomes [44, 52]. A 2022 review evaluated the outcomes of implemented food taxes, showing that food taxes are mostly passed on to consumers and result in reduced sales of taxed foods [52]. Reviews investigating the equity effects of food taxation policies concluded similar or greater effects for socioeconomically disadvantaged populations compared with advantaged populations [46, 52]. One systematic review based on modelling studies found that food taxes are modelled to reduce CVD mortality rate by 8% over a 5 year period (*n*=1 study [89]), while taxes on sugar-sweetened beverages (SSBs) may result in a 1% reduction in CVD cases over a 10 year period (*n*=1 study [90]) [53]. These reviews did not report on cost effectiveness.

#### Fruit and vegetable subsidies

Fourteen systematic reviews report that fruit and vegetable subsidies are associated with increased purchases of fruits and vegetables [2, 44, 45, 52–62]. For instance, a 2024 review found that a price reduction of 20% resulted in a 16.6% increase in fruit and vegetable purchases (95% CI 12.3%, 20.9%) [54]. Few studies focused on health outcomes, but one review reported that in a US population, fruit and vegetable subsidies were modelled to prevent 2690 CVD cases per year (compared with 2690 yearly CVD cases prevented through a national energy labelling law and 45,000 yearly CVD cases prevented through a national salt reduction initiative) [53]. Another reported that lower fruit and vegetable prices were associated with lower body-weight outcomes in low-income children and female individuals [57]. The effects seem to be limited to children and women from socioeconomically disadvantaged families [57, 59, 60, 62]. In addition, it has been found that food subsidy programmes for women from socioeconomically disadvantaged groups are able to effective reduce health inequalities [59]. However, evidence on cost effectiveness of fruit and vegetable subsidies is inconsistent [2]. Most reviews found that subsidies should be at least 10–15% and preferably combined with unhealthy food taxes [45, 54].

#### Bans or restrictions on unhealthy food price promotions

No systematic reviews are available on banning or restricting food price promotions. A recent modelling study suggested that a national mandatory restriction on price promotions of SSBs in Australia could lead to a mean change in daily energy intake of −12.520 kJ (−3 kcal), which would translate to a reduction in body weight of 0.11 kg [91].

#### National level policies on bicycle provision to employees at workplaces

We identified no systematic reviews on the topic of employer bicycle provision policies. Two studies on bicycle provision reported beneficial effects on wellbeing, but no effects on physical activity [92, 93], while there are concerns regarding equity [94–97].

#### Car parking charges in workplaces

No systematic reviews are available on the impact of car parking charges in workplaces. However, free car parking at the workplace has been associated with lower likelihood of active commuting, increased car use, and a reduction in walking, cycling and use of public transport in two original studies [98, 99].

### Cognitive policies

#### Food labelling

Eight systematic reviews summarised the effects of food labelling on purchasing or dietary outcomes [63–70]. Front-of-pack labelling seems to be an effective approach to promote healthier product purchases [63–65], although some of the reviews report mixed efficacy [66, 67]. The type of label investigated seems to matter; for example, the traffic light system has been shown to have smaller effects than the Nutri-Score and nutrient warning labels [64]. Beneficial effects of front-of-pack labelling on dietary intake were reported by one systematic review and meta-analysis [65], based on three experimental studies [100, 101], and physical-activity energy-equivalent food labelling has been shown to reduce mean energy consumption by 334.720 kJ (80 kcal) per day [68]. However, socioeconomically disadvantaged populations may benefit less from food labelling than socioeconomically advantaged populations, highlighting the need to focus on easy-to-understand labelling, which is likely to be effective across populations [69, 70]. Cost effectiveness was not assessed in these eight systematic reviews.

## Discussion of findings

We summarised the evidence for the efficacy, cost effectiveness and equity of policies aimed at preventing or reducing the burden of type 2 diabetes that are both population based and low agency.

For the majority (11 out of 16) of population-based, low-agency policies, systematically reviewed evidence on their effectiveness was available. Eight of these 11 policies suggested favourable effects on lifestyle behaviours and, occasionally, on health outcomes, and no negative effects were reported. Substantial evidence suggests that fruit and vegetable subsidies, unhealthy food taxes, mass media campaigns and school nutrition and physical activity education are effective in promoting healthier lifestyle behaviours. Only physical activity education can be labelled as a cost-effective policy based on the current evidence, with contradictory results for mass media campaigns and fruit and vegetable subsidies; there was no evidence on cost effectiveness for the remaining policies. Furthermore, no systematically reviewed evidence was available for policies on zoning, mandatory healthy checkouts, bans or restrictions on unhealthy food price promotions, provision of bicycles to employees at workplaces and workplace car parking charges.

The lack of meta-analysed policy effects was notable. This is likely due to the heterogeneity in exposures and outcomes, and the limited number of studies conducted on each specific exposure–outcome association (i.e. many policies have only been implemented or evaluated in a couple of instances). This lack of quantitative results makes it difficult to summarise the expected effect size of the different policies. One review summarising modelling studies on mandatory food reformulation concluded that a 5% and 30% reduction in sugar content could reduce obesity prevalence by 0.2% to 0.9%, respectively, and type 2 diabetes incidence by 5.8 and 31.1 cases per 100,000 persons-years, respectively [81]. A review on fruit and vegetable subsidies found that a price reduction of 20% resulted in a 16.6% increase in fruit and vegetable purchases [54], while a meta-analysis suggested that year-long school nutrition and physical activity lessons could lower BMI *z* scores by 0.14 [26]. These effect size are all relatively small but population-based policies reach the whole population, which likely leads to larger effects at a population level than approaches that only reach a high-risk population [10]. Nevertheless, as also reiterated by several of the included reviews (e.g. [53]), it is important to combine several policies to achieve a substantial impact on the prevalence and incidence of obesity and type 2 diabetes.

There was an overall lack of evidence on the equity effects of the considered policies; evidence was available for only five out of 11 policies for which systematically reviewed evidence was available. Reviews that did report on equity concluded mostly that the population-based, low-agency policies had favourable equity effects, with the exception of food-labelling policies. It is important to note that we classified policies as ‘low agency’ when they were characterised by ‘passive exposure’. As such, food-labelling policies were included as low-agency policies, despite the fact that they may require individual resources, such as nutritional knowledge and financial resources (i.e. active engagement) in order to benefit from them. Food labelling could also be considered as ‘high agency’ if the level of engagement, rather than the level of exposure, was considered in the dichotomisation. Whether food-labelling policies should be regarded as low or high agency also depends on the type of food labelling, whereby front-of-pack colour schemes may be less agentic and potentially more equitable than food labels with written nutritional information [69].

Policies that are characterised by passive exposure as well as passive engagement (i.e. ones for which recipients do not have to change their routines or be aware of the policy to benefit) are arguably most equitable because behaviour change could be achieved and maintained via minimal personal effort and, thus, health effects can be maximised. However, very limited evidence was available for policies with passive engagement, which included those targeting mandatory food reformulation, bans or restrictions on unhealthy product marketing, restricted availability of unhealthy food and reduced portion size of unhealthy products. The available evidence suggested that these policies were effective in reducing unhealthy purchases and energy intake [36–42], but did not report on equity effects. This remains an important avenue for future research.

Real-world evidence on the equity effects of population-based policies is especially welcome since much of the evidence around equity has been derived from controlled experimental settings or modelling studies. Evidence on the differential impact of the Mexico SSB tax suggests that this tax most effectively decreased SSB purchases in low-income households [102]. Yet, as reported in this narrative review, the effects of food taxes are generally stronger in low- and middle-income countries than in high-income countries [47, 48]. It is also important to note that, overall, evidence mainly originated from Western, educated, industrialised, rich and democratic (WEIRD) countries, and future research should consider whether the impact of policies are similar in non-WEIRD countries [103].

Very few reviews reported on health outcomes, such as weight loss or reductions in type 2 diabetes risk. A systematic review on food reformulation modelling studies found that reductions in the sugar content of foods and beverages of 5–100% could reduce obesity prevalence and/or type 2 diabetes incidence, although available evidence was limited [38]. Indeed, the time lag between exposure to a population-based policy and impact on disease outcomes is substantial; thus, detecting the effects of these policies on disease (risk factors) requires a very long time frame and sustained behaviour change in a real-world setting, or a simulation study modelling the health effects of the policies [104].

It remains to be decided what evidence would best support the implementation of population-based policies. The evidence summarised in this narrative review stems from a range of different study designs. Evidence from trials in controlled settings, such as that available for food reformulations policies, benefits from high internal validity due to the lack of confounding bias and high control over the treatment timing and assignment. However, translation to the real world may be hampered by significant selection bias and the absence of external influences; this is also referred to as the efficacy–effectiveness gap [105]. Moreover, in the real world, it is mostly impossible, unethical or unaffordable to randomise entire areas into policy adoption or not to evaluate effectiveness. It has been speculated that the lack of real-world evidence is hampering the implementation of type 2 diabetes prevention policies [106]. Therefore, alternative designs are needed to provide such evidence. For some policies, evidence has mainly been derived from modelling studies, which also lack the nuances of real-world situations and rely on data from observational and experimental studies to inform model assumptions. Nevertheless, modelling studies can help address research questions, including those about long-term health effects, which no single study can address. Natural experiments may also offer an alternative study design, whereby random allocation to an event or exposure is assumed and researchers compare behavioural or health outcomes between the exposed and unexposed groups [107]. The plausibility of the as-if randomisation of the exposure is crucial for this study design and should be investigated both quantitatively and qualitatively to strengthen the causal inference of the natural experiment [108]. In addition, causal frameworks, such as the target trial emulation, can be used to strengthen the causal inference based on observational data [109]. However, it is unlikely that any single method will provide definitive evidence. As such, triangulation of different sources of evidence should be pursued to provide a solid basis for policy recommendations [110, 111]. For instance, evidence on the effectiveness of food taxes comes from modelling studies, evaluations of natural experiments and observational studies from various countries and is combined with modelling studies of the effects of these policies on obesity rates, while the evidence on reduced portion size policies originates mainly from RCTs.

Nevertheless, there is a fine balance between promoting policies with unproven effectiveness and not implementing policies because we are waiting for high certainty of evidence that may never be available. The current evidence base did not permit for the comparison of strength of evidence across policies given the lack of reporting on risk of bias in many reviews and heterogeneity in the study designs applied. Moreover, the medical-evidence grading system may not be suitable for the population-based policies under study here. During the coronavirus disease-2019 (COVID-19) crisis, policies on social distancing and mask wearing were implemented without robust, high-quality evidence [112], under the assumption that they would do more good than harm. First, this may suggest that research into population-based policies should focus much more on their potential harms [113]. Second, research should focus on the role of evidence in the policy-making process [114], such as theorised in the Multiple Streams Framework [115] and the Punctuated Equilibrium Theory [116].

In conclusion, there is a large evidence base for the effectiveness of population-based, low-agency policies for the prevention of type 2 diabetes. However, the quality of the evidence is variable, and whether these low-agency policies are indeed equitable remains uncertain given the lack of evidence. There is clear evidence for beneficial effects for fruit and vegetable subsidies, unhealthy food taxes, mass media campaigns and school nutrition and physical activity education. Meanwhile, limited evidence is available on cost-effectiveness and equity effects, but the available evidence points towards mainly equitable effects. Future studies should prioritise triangulation of different sources of evidence to provide a solid evidence base for policy recommendations. Given the relatively small effect sizes reported, it is advisable to combine different policy measures to maximise the reduction in the burden of type 2 diabetes.

## Supplementary Information

Below is the link to the electronic supplementary material.

Slideset of figures (PPTX 319 KB)

## Abbreviations

SSB: Sugar-sweetened beverage
WEIRD: Western, educated, industrialised, rich and democratic
