# Supplementary material for: Evidence on the effectiveness and equity of population-based policies to reduce the burden of type 2 diabetes: a narrative review
Source: Diabetologia. 2024 Dec 2;68(2):281–94. doi: 10.1007/s00125-024-06330-1 (PMC11732888; doi:10.1007/s00125-024-06330-1)
Supplement: Supplementary file 1 — Slideset of figures (PPTX 319 KB) [file 125_2024_6330_MOESM1_ESM.pptx]

## Slide 1
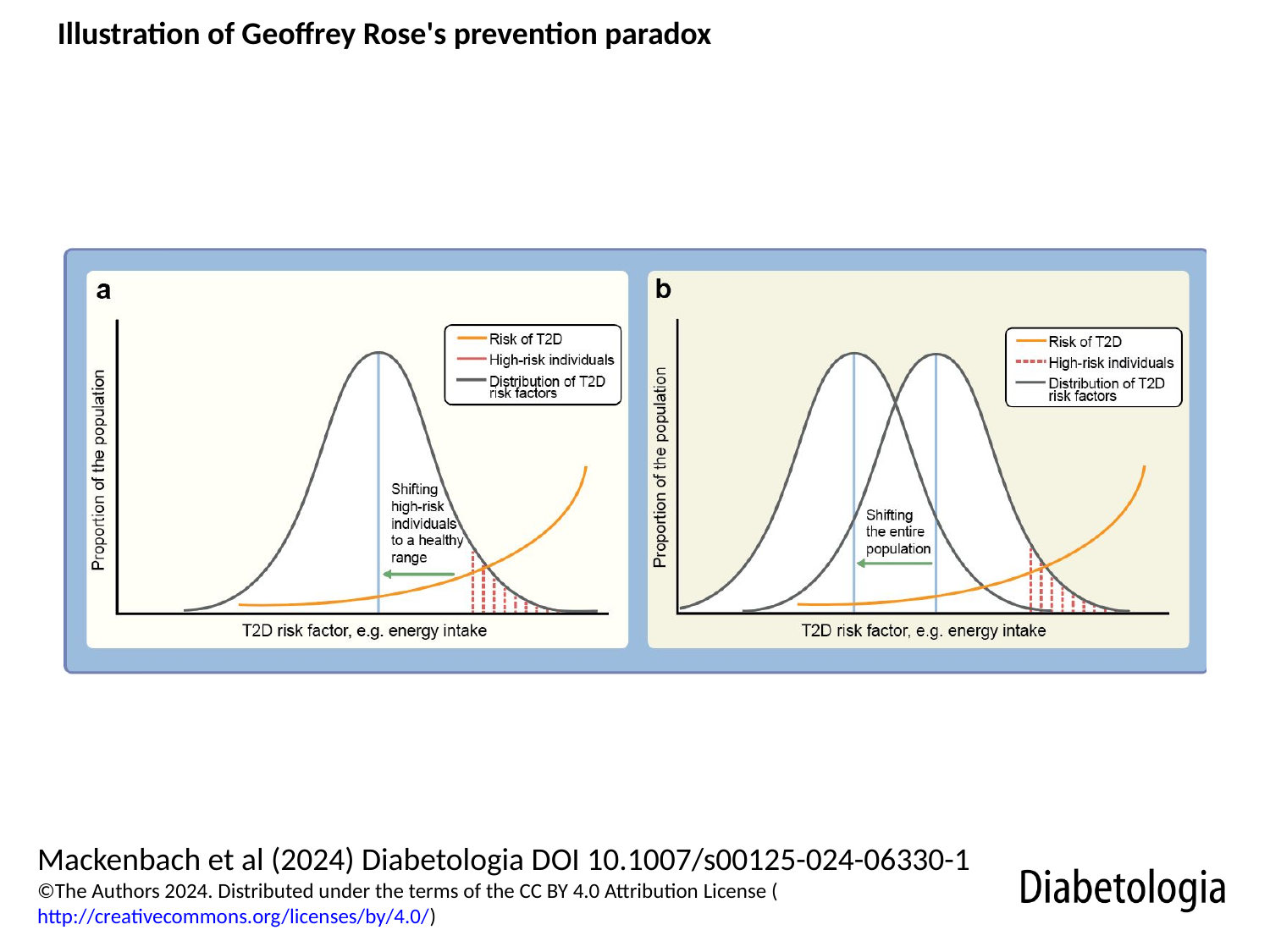

Illustration of Geoffrey Rose's prevention paradox
Mackenbach et al (2024) Diabetologia DOI 10.1007/s00125-024-06330-1
©The Authors 2024. Distributed under the terms of the CC BY 4.0 Attribution License (http://creativecommons.org/licenses/by/4.0/)

## Slide 2
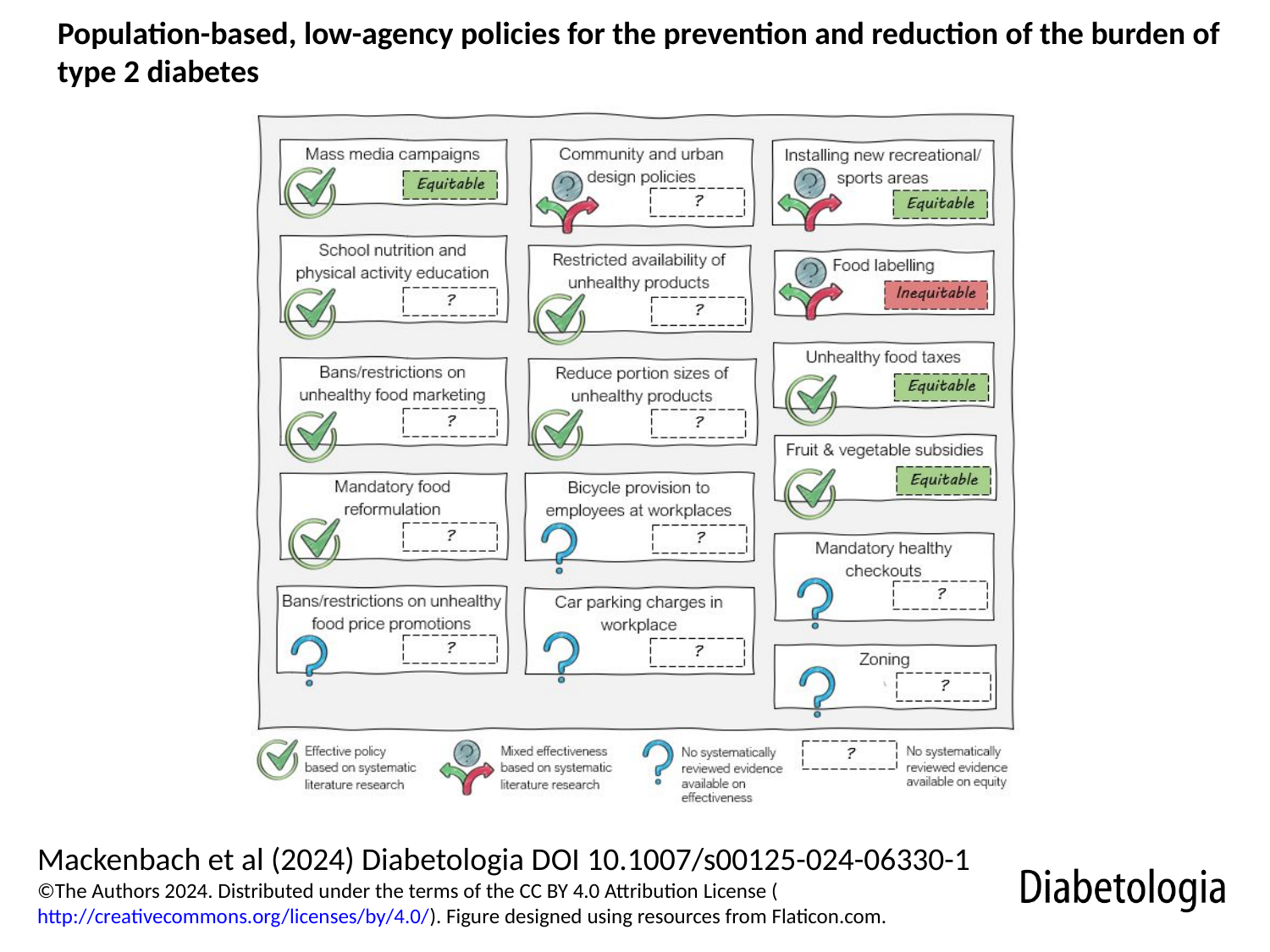

Population-based, low-agency policies for the prevention and reduction of the burden of type 2 diabetes
Mackenbach et al (2024) Diabetologia DOI 10.1007/s00125-024-06330-1
©The Authors 2024. Distributed under the terms of the CC BY 4.0 Attribution License (http://creativecommons.org/licenses/by/4.0/). Figure designed using resources from Flaticon.com.
